# Data on characterizing the gene expression patterns of neuronal ceroid lipofuscinosis genes: CLN1, CLN2, CLN3, CLN5 and their association to interneuron and neurotransmission markers: Parvalbumin and Somatostatin

**DOI:** 10.1016/j.dib.2016.06.027

**Published:** 2016-06-23

**Authors:** Helena M. Minye, Anna-Liisa Fabritius, Jouni Vesa, Leena Peltonen

**Affiliations:** a Department of Applied Biology, University of Helsinki, Finland; b Department of Human Genetics, David Geffen School of Medicine, University of California, Los Angeles, CA, USA; c Department of Molecular Medicine, Biomedicum, National Public Health Institute, PO Box 104, FIN 00300 Helsinki, Finland

**Keywords:** Neurogenetics, CLN genes, CLN1/CLN2/CLN3/CLN5, Embryonic neurogenesis, Neurotransmission, molecular genetics, Neuronal ceroid lipofuscinoses, Neuronal differentiation, neuroplasticity, and synaptic pruning

## Abstract

The article contains raw and analyzed data related to the research article “Neuronal ceroid lipofuscinosis genes, CLN2, CLN3, CLN5 are spatially and temporally co-expressed in a developing mouse brain” (Fabritius et al., 2014) [1]. The processed data gives an understanding of the development of the cell types that are mostly affected by defective function of CLN proteins, timing of expression of CLN1, CLN2, CLN3 and CLN5 genes in a murine model. The data shows relationship between the expression pattern of these genes during neural development. Immunohistochemistry was used to identify known interneuronal markers for neurotransmission and cell proliferation: parvalbumin, somatostatin subpopulations of interneurons. Non-radioactive in-situ hybridization detected CLN5 mRNA in the hippocampus. Throughout the development strong expression of CLN genes were identified in the germinal epithelium and in ventricle regions, cortex, hippocampus, and cerebellum. This provides supportive evidence that CLN1, CLN2, CLN3 and CLN5 genes may be involved in synaptic pruning.

**Specifications Table**

**Table:** 

Subject area	Molecular Biology, Molecular Pathology
More specific subject area	Molecular Genetics, Molecular Medicine, Neurogenetics, Neurogenesis
Type of data	Images, microscopy, figures
How data was acquired	Microscopy, immunohistochemistry, molecular biology, murine models, non-radioactive in-situ hybridization.
Data format	Raw, analyzed
Experimental factors	For in-situ analyses, sectioned, fixation in 4% paraformaldehyde. For immunohistochemistry, brains and embryos were post-fixed in 4% PFA, embedded in paraffin.
Experimental features	The timing of genetic expression of CLN1, CLN2, CLN3 and CLN5 genes were investigated during brain development. Tested murine model with known interneuronal markers using immunohistochemistry.
Data source location	Los Angeles, California, United States. Geographic coordinates 34.0722°N, 118.4441°W
Data accessibility	Within the Data in Brief article.

**Value of the data**

• CLN 2, CLN3, CLN5 genes are expressed in the glia, hippocampus, cerebellum, and ventricles in a murine model can be functionally categorized.
• Specific evidence of genetic expression of CLN2, CLN3, CLN5 give insights to the selective neurodegeneration known in the disease.
• CLN1, CLN2, CLN3 and CLN5 gene expression profiles play an important role in early embryonal neurogenesis; may stimulate further research in their role in neuronal differentiation, neuroplasticity, and synaptic pruning.

## 1 Data

Neuronal ceroid lipofuscinosis genes: CLN1, CLN2, CLN3, CLN5 were examined in neuronal tissue during mouse brain development. Interneuronal subpopulations immunoreactive to parvalbumin and somatostatin were identified to be co-expressed with CLN1, CLN2, CLN3, CLN5 proteins in the postnatal cortex and hippocampus.

## 2 Experimental design, materials and methods

### 2.1 Tissue preparation

**Fig. 1:**
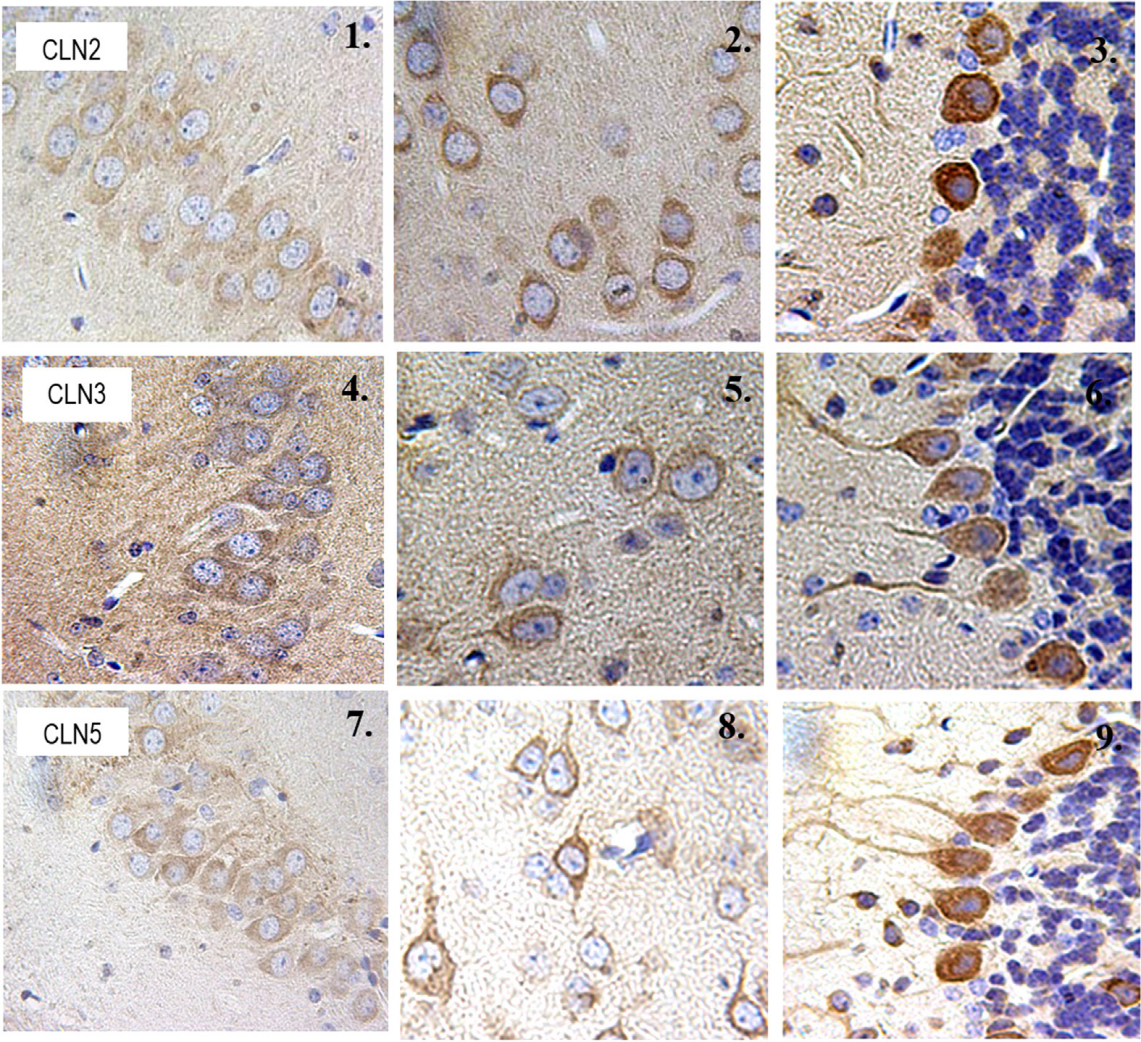
CLN2, CLN3, and CLN5 expression in the hippocampus, cortex and cerebellum in P24. 1-1: Immunodetection of CLN2 in hippocampus. 1-2: Immunodetection of CLN2 in cortex. 1-3: Immunodetection of CLN2 in Purkinjie cells. 1-4: Immunodetection of CLN3 in hippocampus. 1-5 Immunodetection of CLN3 in cortex. 1-6: Immunodetection of CLN3 in Purkinjie cells. 1-7: Immunodetection of CLN5 in hippocampus. 1-8: Immunodetection of CLN5 in cortex. 1-9: Immunodetection of CLN5 in Purkinjie cells. CLN3 and CLN5 proteins were strongly expressed in the inner migratory zone. Positive signaling is indicative by the brown stain, blue is the counterstain.

**Fig. 2:**
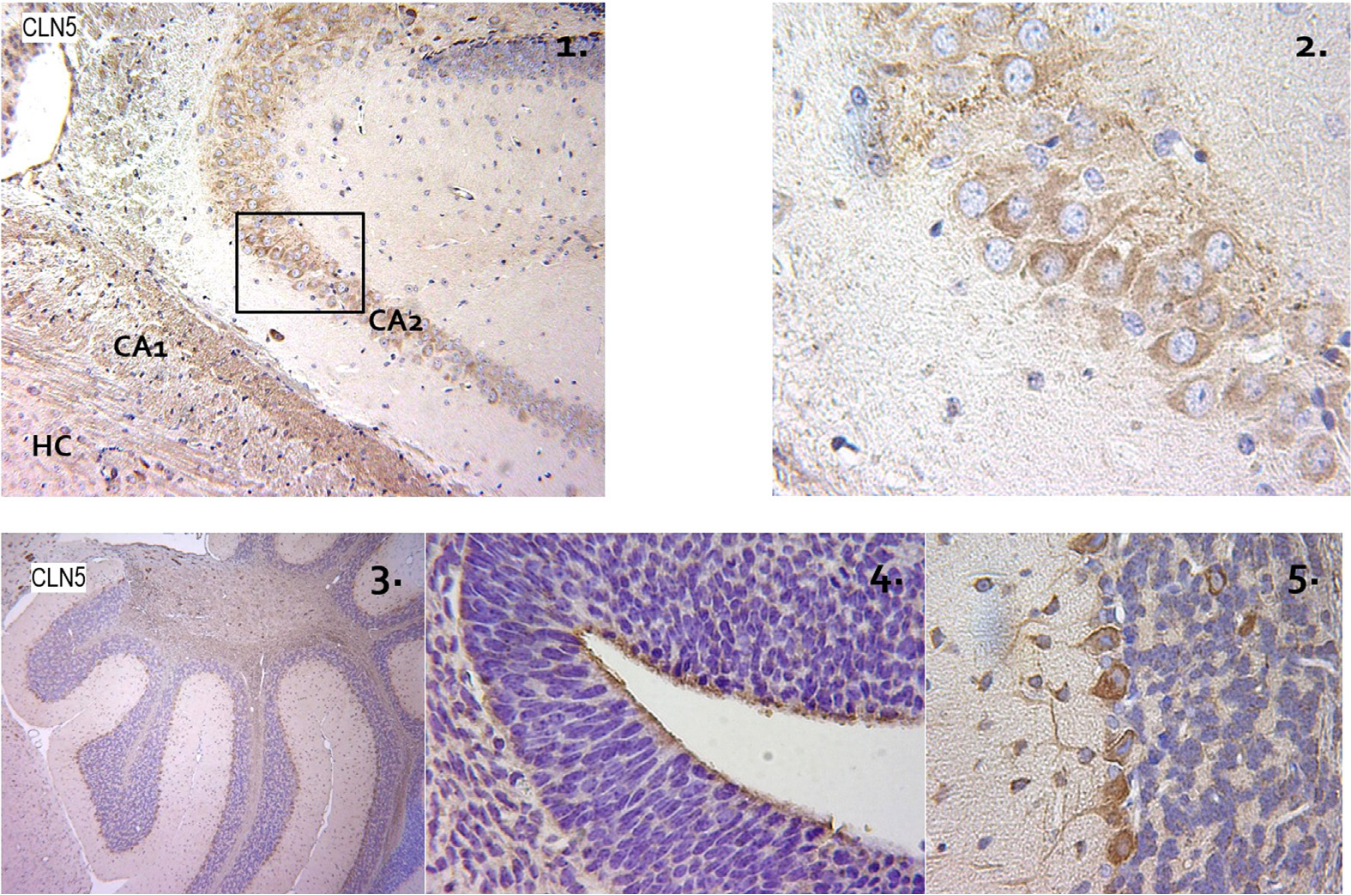
Expression pattern of CLN5 in developing E18.5 hippocampus and cerebellum. 2-1: Hippocampus layers, CA1 and CA2. 2-2: Magnified view [100×] of pyramidal cells in selected region of 1. Positive signaling is indicative by the brown stain, blue is the counterstain. 2-3: Developing cerebellum, 2-4: Magnified view [100×] of the developing cerebellum. 2-5: Magnified [400×] Purkinjie cell layer stained with CLN5 antibody in embryonic cerebellum.

**Fig. 3:**
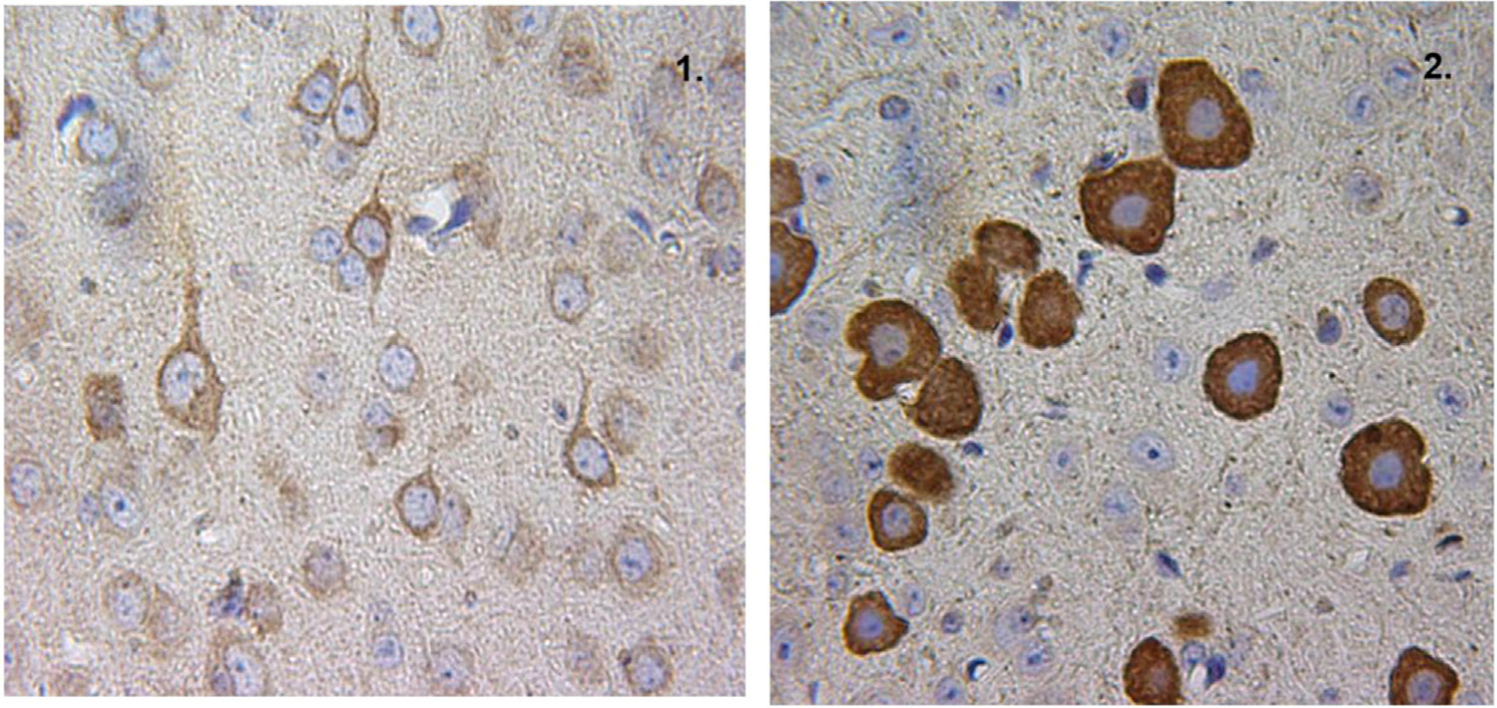
3-1: CLN5 protein detected in the axon and dendrites of neurons in the cortex in P24 mouse brain. 3-2: Intense immunopositivity of CLN5 protein in the cytoplasm of interneurons in pons in P24 mouse brain. Positive signaling is indicative by the brown stain, blue is the counterstain.

**Fig. 4:**
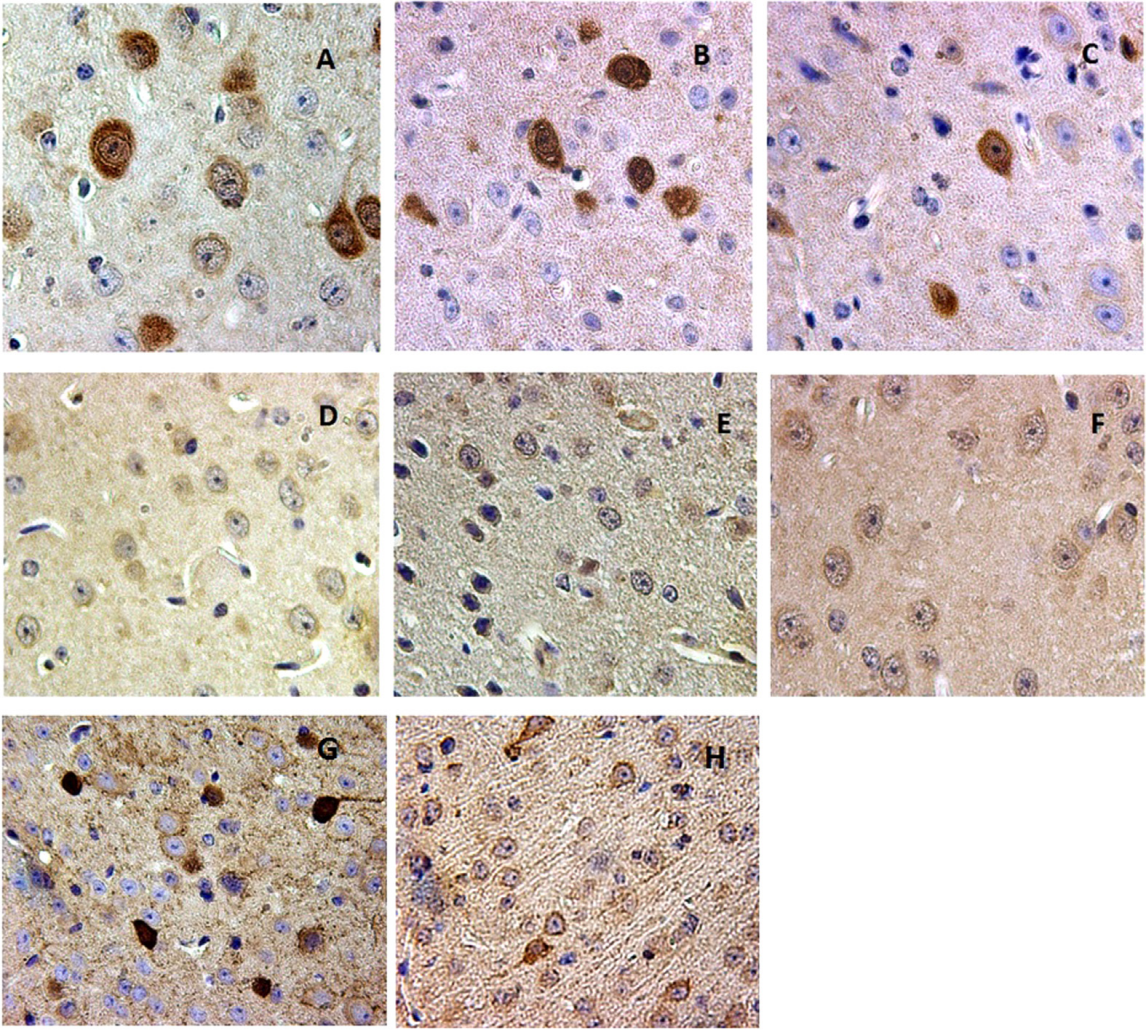
CLN1, CLN2, CLN3, CLN5 expressed in parvalbumin and somatostatin subpopulations of interneurons in P60 mouse cortex. 4-A: CLN2 immunopositivity in parvalbumin-type interneurons. 4-B: CLN3 immunopositivity in parvalbumin-type interneurons. 4-C: CLN5 immunopositivity in parvalbumin-type interneurons.4-D: CLN2 immunopositivity in somatostatin-type interneurons. 4-E: CLN3 immunopositivity in somatostatin-type interneurons. 4-F: CLN5 immunopositivity in somatostatin-type interneurons. 4-G: CLN1 immunopositivity in parvalbumin-type interneurons. 4-H: CLN1 immunopositivity in somatostatin-type interneurons. Positive signaling is indicative by the brown stain, blue is the counterstain.

**Fig. 5:**
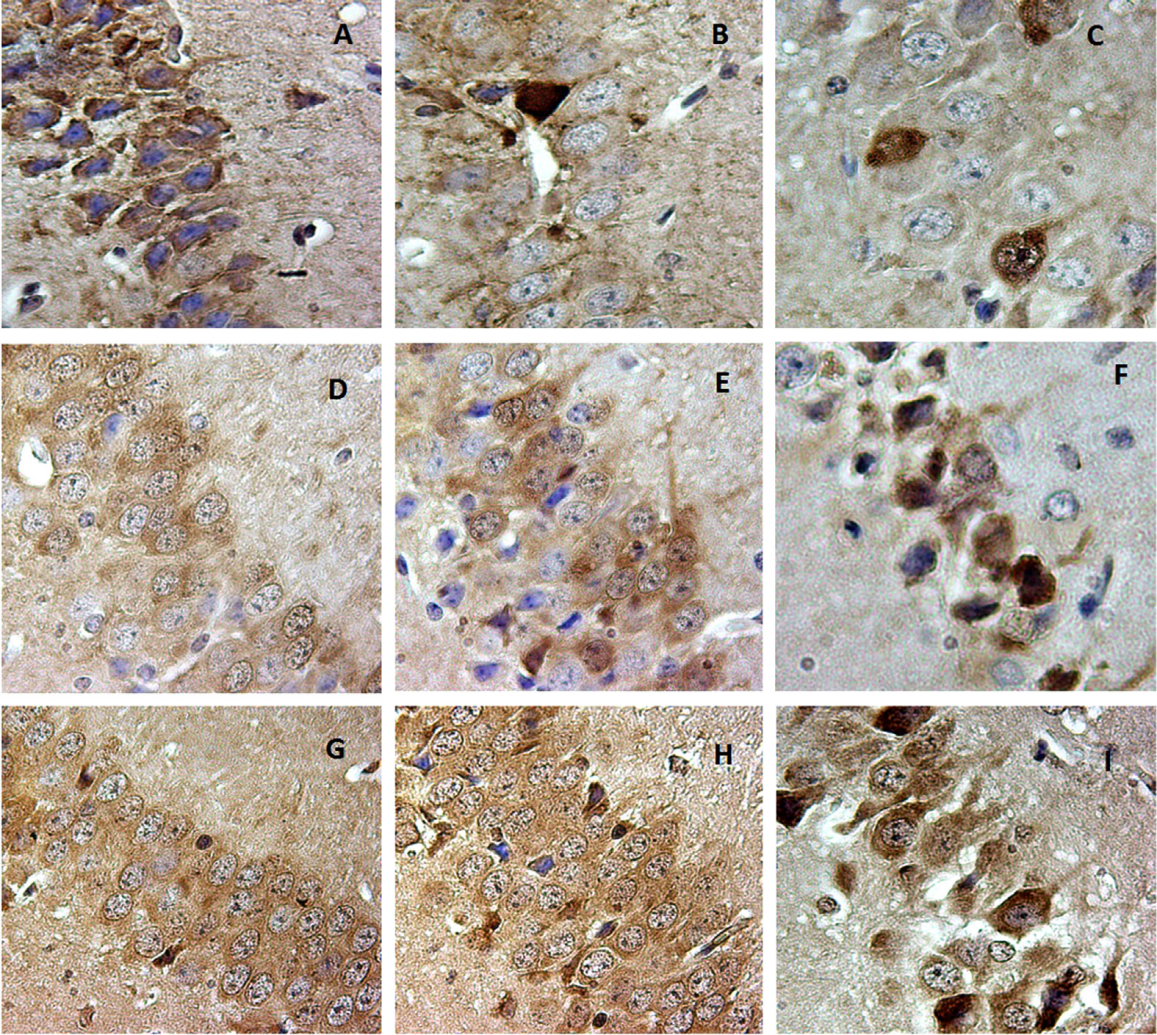
Immunopositive for reactivity of CLN2, CLN3, CLN5 in somatostatin subpopulation of interneurons in the hippocampus in P24. 5-A, B, C: CLN2 expressed in somatostatin positive cells in the hippocampus. 5-D, E, F: CLN3 expressed in somatostatin positive cells in the hippocampus. 5-G, H, I: CLN5 expressed in somatostatin positive cells in the hippocampus. Positive signaling is indicative by the brown stain, blue is the counterstain.

Brains and embryos were obtained from mouse strain C57 BL/6J on embryonic days E18.5 and postnatal days P24 and P60. The morning of the vaginal plug was defined as embryonic day 0.5 (E0.5) and the day of birth as P0. Animals were maintained in specific pathogen-free conditions according to the regulations of the Department of Laboratory Animal Medicine at the University of California, Los Angeles. Animals were anesthetized of animals was performed with 0.04 ml of ketamine HCl (25 mg/ml, Ketaject, Phoenix Scientific, Inc., St. Joseph, MD, USA) after which they were sacrificed by cervical dislocation. For immunohistochemistry, older animals (P24 and P60) were intracardially perfused with 4% paraformaldehyde (PFA) in 0.1 M phosphate buffered saline (PBS) before removal of the brain [1]. All procedures were performed according to institutional and NIH guidelines (Figs. 1–5).

### 2.2 Reverse transcription

CLN5 RNA was isolated from mouse brain tissue RT-PCR was converted to cDNA with 9961(1:30) primers: 3′CACTGGAAGGAAAACGGGACA 5′ and S5513A04 (1:10): 5′ GGGCCCAAAGGAAACAAG 3′ and 9960 (1:30) primer using Superscript reverse transcriptase and 10× dNTPs incubated at 45 °C for 50 min. All probes are specific based on NCBI matches. The cDNA was then amplified with Pfu DNA Polymerase and reamplified Taq DNA polymerase (Amersham Pharmacia Biotech). The DNA template was then purified with Qiaquick PCR purification to remove the enzymes, excess dNTPs and polymerases, and salts.

### 2.3 Transformation

*Escherichia coli* cells were transformed using pGEM-T Easy Vector that contains a T7 and SP6 promoter on each end of the MCS (Promega). Using 2.5 µl of DNA template obtained as PCR product and T4 DNA Ligase (3 Weiss units/µl) were used for a total reaction of 10 µl. Cells went through heat shock stress and screened using IPTG and Lac Z expression after plating on LB and 50 µg/ml Ampicillin overnight at 4 °C for four of the six ligations and at 37 °C for the remaining two. Transformants were isolated using blue/white screening. Single colonies were inoculated in LB-amp media at 37 °C. To determine whether the insert was cloned, the insert was removed using NotI cleavage from the plasmid and run on a 1% high melt agarose gel. Plasmids were recovered by maxiprep (Qiagen).

### 2.4 Sequencing

**Fig. 6:**
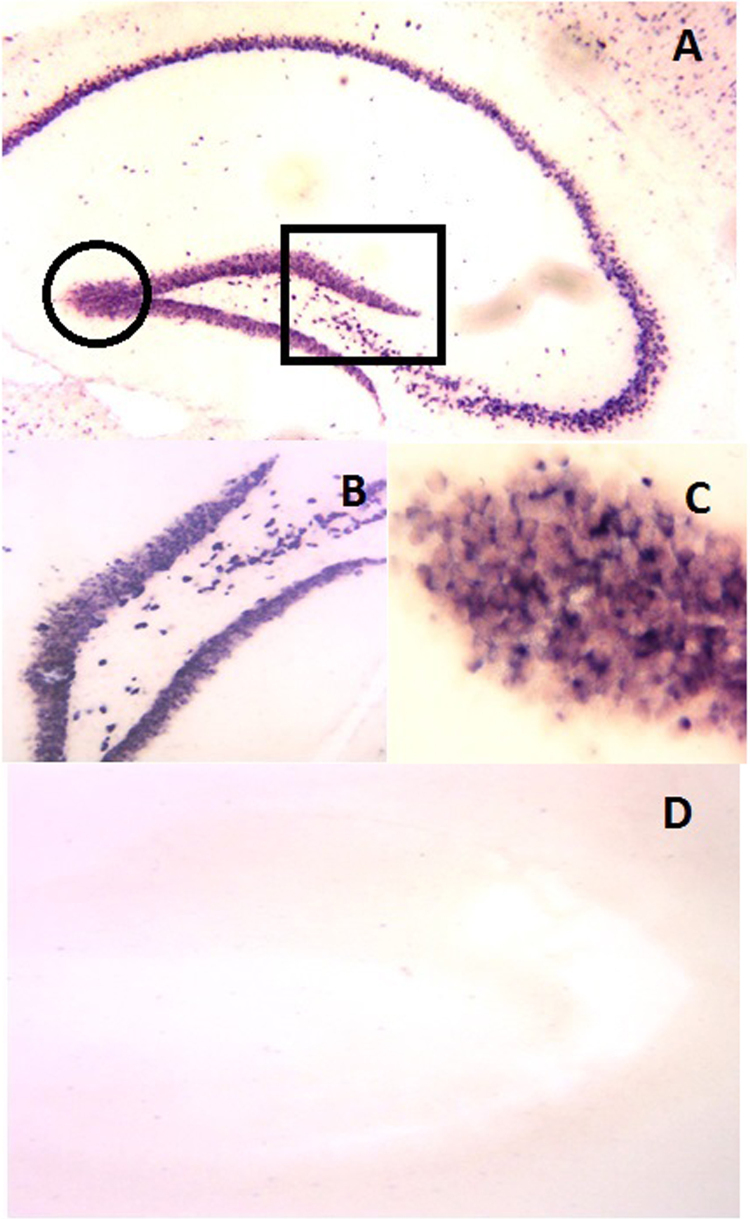
CLN 5 expression of RNA antisense DIG-labeled transcripts in the longitudinal section of the P60 hippocampus. 6A: mRNA expression of CLN5 in mouse brain at 60 days can be observed in the hippocampus. 6B: Magnified [100×] mRNA expression of CLN5 in mouse brain at postnatal 60 days. 6C: Magnified [400×] mRNA expression of CLN5 in mouse brain at postnatal 60 days. 6D: Negative control for specificity of the CLN5 probe.

DNA was precipitated by ethanol treatments and sodium acetate pH 4.6. BigDye was used sequencing to determine if both sense and antisense strands have be cloned as well as the orientation at insertion in the plasmid. Single restriction digest with EcoRI was not useful in determining insert orientation because it resided approximately in the middle of the insert (223 bp/336 bp); making the orientation of insert as well as the sequence identifiable using Sequencher (Fig. 6).

### 2.5 Non-radioactive probe synthesis

The plasmid was linearized with SpeI and NcoI. The SpeI digest and NcoI digested templates were transcribed with T7 RNA polymerase and SP6 polymerase (with additional 100 µM MgCl2 added) respectively to generate sense ssRNA and NTP mix the digoxigenin labeled uracil incorporated into the RNA by in vitro transcription (Roche Molecular Biochemicals). RNA was precipitated using ethanol and 4 M lithium chloride. The RNA was quantitated using dot-blot technique on nylon membrane and immunodetected with Anti-digoxigenin Fab fragments (Roche Molecular Biochemicals) conjugated with alkaline phosphatase and identified with substrate, NBT/BCIP (Roche Molecular Biochemicals). Presence of RNA was also confirmed on a 1% high melt agarose gel.

### 2.6 Non-radioactive in-situ analyses

Brain sections at varying stages of development were sectioned in paraffin. Sections were washed with glycine in 0.1 M phosphate buffer, 0.1 M phosphate buffer, followed by 0.25% acetic anhydride in 0.1 triethanolamine (TEA) pH 8.0, and dehydrated using ethanol and chloroform. Hybridization preparation included 300 µg/ml salmon sperm, 15 µg/ml tRNA, 40 mM DTT, and digoxgenin labeled probe diluted in hybridization buffer and incubated at 60 °C overnight with DPX mountant. The sense and antisense transcripts were then washed with decreasing SSC concentration and blocked with 0.05 g BSA/10×TBS at 37 °C. Section were incubated with 1:500 Anti-digoxigenin Fab fragments (Roche Molecular Biochemicals) in 4 °C conjugated with alkaline phosphatase and identified with substrate, NBT/BCIP (Roche Molecular Biochemicals) in detection buffer pH 9.5. Then dehydrated with ethanol and HemoD (Fig. 6).

### 2.7 In-situ hybridization probes

templates for the probes for in situ hybridization were generated from mouse brain total RNA by reverse transcriptase (RT)-PCR amplification (Invitrogen, Carlsbad, CA). CLN2 probe was synthesized using a CLN2-specific sense primer (nucleotides 575–593 in mouse cDNA) and an antisense primer (nucleotides 878–856 in mouse cDNA). CLN3 probe was obtained using a CLN3-specific sense primer (nucleotides 40–60 in mouse cDNA) and an antisense primer (nucleotides 370–349 in mouse cDNA). CLN5 probe was amplified from mouse cDNA that corresponded nucleotides 1606–1803 in human CLN5 cDNA. Primers used for amplification of CLN5 were 5′-TACAAGCGCTTCTCTTTCCGTC-3′ (sense-primer) an ATCCCACGGCGTCATGCATAAGTTT-3′ (antisense-primer) [7]. PCR-amplified cDNA fragments were cloned into pGemT-Easy (Promega, Madison, WI). Images were taken with Olympus digital camera and minimally processed in Adobe Photoshop Elements program (Figs. 1–5).

### 2.8 Antibodies

To obtain mouse CLN5 protein for immunization, the cDNA of mouse CLN5, corresponding to human cDNA nucleotides 1606–2520, was cloned into a pGEX-6P-1 vector (Pharmacia, Sweden). The CLN5 polypeptides were expressed in the *E. coli* strain BL21-E as a glutathione S-transferase (GST) fusion protein and purified with Glutathione Sepharose (Pharmacia, Sweden). Rabbits were immunized by subcutaneous injection with 500 μg of the CLN5 fusion protein in Freund׳s complete adjuvant. Immunization was repeated four times and the blood was collected one week after the last immunization [1]. A small sample of blood was also collected before the first immunization (i.e. pre immune serum). The CLN2-specific antibody against amino acids 368–383 in human CLN2 and the antibody against the mouse CLN3 peptide have been previously described [2], [4], [5], [6].

### 2.9 Immunohistochemistry

Brains and embryos were post-fixed in 4% PFA for 20 h after which they were embedded in paraffin. The tissue was cut into 4 μm sections placed on Superfrost Plus slides. Immunohistochemical staining: after deparaffinization with xylene and decreasing graded series of ethanol solutions, endogenous peroxidase activity in mouse tissue sections was blocked with 3% H2O2 in methanol for 10 min. For antigen retrieval, sections were incubated in 10 mM Na-citrate buffer (pH 6.0) at 95 °C for 20 min followed by three washes (5 min each) in PBS-buffer. Unspecific binding of antibodies was blocked by incubating sections in 3% goat normal serum (Vector, Burlingame, CA) in PBS for 30 min after which avidin–biotin blocking was performed according to manufacturer׳s protocol (Vector). Primary antibodies were diluted in 3% goat normal serum: CLN1 antibody in 1:300 [8], CLN2 antibody in 1:500, CLN3 antibody in 1:250 and CLN5 antibody in 1:1000 dilutions. Parvalbumin-specific antibodies (Swant 1:6000 dilution) [3] and somatostatin-specific antibodies (Swant 1:5000). Primary antibodies were incubated for 18 h at 4 °C after which sections were washed three times for 5 min in PBS buffer followed by incubation in secondary antibody, goat anti-rabbit antiserum (1:200), for 30 min at RT. After 3×5 min rinses in PBS, sections were treated with avidin-biotinylated horseradish peroxidase complex (ABC, Vector) for 30 min at room temperature followed by three rinses in PBS (5 min each). Binding of antibodies was detected with DAB staining kit (Vector) after which the sections were counter stained in Harris Modified Hematoxylin (Fisher), dehydrated in increasing series of ethanol and xylene and mounted with mounting medium (Richard Allan Scientific). Images were captured with an Olympus microscope and a camera. As a control for specificity of immunosera, pre-immune serum was used when available (for CLN2 and CLN5). For CLN5, as a control for unspecific staining, blocking of antigenic sites with CLN5 protein was also used [2], [7]. This was done by incubating 5–25 times excess of CLN5 protein together with CLN5 immunosera at RT for 30 min. This mixture was then used along the immunostaining procedure as a primary antiserum. The incubation without the primary antibody served as a general control [1].
